# De novo Nanopore read quality improvement using deep learning

**DOI:** 10.1186/s12859-019-3103-z

**Published:** 2019-11-06

**Authors:** Nathan LaPierre, Rob Egan, Wei Wang, Zhong Wang

**Affiliations:** 1 Department of Computer Science, University of California, Los Angeles, Los Angeles, CA, 90095 USA; 2 Department of Energy Joint Genome Institute, Walnut Creek, CA, 94598 USA; 3 EGSB Division, Lawrence Berkeley National Laboratory, Berkeley, CA, 94720 USA; 4 School of Natural Sciences, University of California at Merced, Merced, CA, 95343 USA

**Keywords:** Deep learning, Long sequence reads, Oxford Nanopore, de novo assembly

## Abstract

**Background:**

Long read sequencing technologies such as Oxford Nanopore can greatly decrease the complexity of de novo genome assembly and large structural variation identification. Currently Nanopore reads have high error rates, and the errors often cluster into low-quality segments within the reads. The limited sensitivity of existing read-based error correction methods can cause large-scale mis-assemblies in the assembled genomes, motivating further innovation in this area.

**Results:**

Here we developed a Convolutional Neural Network (CNN) based method, called MiniScrub, for identification and subsequent “scrubbing” (removal) of low-quality Nanopore read segments to minimize their interference in downstream assembly process. MiniScrub first generates read-to-read overlaps via MiniMap2, then encodes the overlaps into images, and finally builds CNN models to predict low-quality segments. Applying MiniScrub to real world control datasets under several different parameters, we show that it robustly improves read quality, and improves read error correction in the metagenome setting. Compared to raw reads, de novo genome assembly with scrubbed reads produces many fewer mis-assemblies and large indel errors.

**Conclusions:**

MiniScrub is able to robustly improve read quality of Oxford Nanopore reads, especially in the metagenome setting, making it useful for downstream applications such as de novo assembly. We propose MiniScrub as a tool for preprocessing Nanopore reads for downstream analyses. MiniScrub is open-source software and is available at https://bitbucket.org/berkeleylab/jgi-miniscrub.

## Background

Long read sequencing has become increasingly important in recent years, with sequencing technologies from companies such as Pacific Biosciences [1] and Oxford Nanopore [2] seeing wide use in a variety of applications including genome assembly [1, 3], detection of antimicrobial resistance genes [4], sequencing personal transcriptomes [5], and improving draft genomes [6]. Genome assembly is one of the most promising and widely-explored of these applications, as long repeat sections have been shown to be among the most important factors that affect assembly quality [7, 8], and long sequencing reads are much more capable of resolving these long repeats. Theoretical analysis has indicated that increasing read length from 100bp to 1000bp significantly simplifies the *de Bruijn* graphs used in assembly algorithms and can increase N50 size by six folds [7].

However, current single molecule, long sequencing reads also have very high error rates, ranging from 5 to 40% [3] per read and often average about 10 to 20% [1, 9], depending on variables such as the type and version of the sequencing technology and the experiment being performed. These high error rates can confound assembly and other analysis and introduce significant computational burdens [2, 3, 9, 10]. It is thus critical that methods be developed towards addressing this issue so that the potential of long read sequencing can be fully realized. Many current solutions involve “hybrid error correction” [3, 9, 11] by performing an additional sequencing run using low-error short reads and aligning them to the long reads, followed by a consensus approach to produce the correct sequence. Despite their success [3, 9, 11], the requirement for extra sequencing runs, often with different technologies, imposes additional monetary and temporal burdens [12]. Another approach involves re-analyzing the raw signal output by the sequencing machines to call the correct bases in the reads [13, 14], but researchers may want not always have this raw signal data available [15].

Thus, it is desirable to have a de novo method for improving long sequencing reads that does not rely on any information other than the reads themselves and is generally applicable across many technologies. Gene Myers [16] and others [3] observed that long read errors tend to locally cluster into certain low-quality “junk” segments, raising the possibility of “scrubbing” [16] (removing) these low-quality segments to significantly improve read quality. We use this term to avoid confusion with the similar term “trimming”, which is usually used to refer to removing adapters and low quality bases primarily off of the *ends* of short reads [17, 18]. Recent work has addressed a related problem of de novo read error correction [19, 20]. However, even the best methods still produce quite a few mis-assemblies, suggesting that independent and complementary methods are necessary for further improving assembly results. Additionally, most of these methods are developed for the single genome setting, and may not perform well in the metagenome setting.

Here we describe MiniScrub, a method for long Nanopore read scrubbing. MiniScrub performs read-to-read overlapping and converts this information into images, followed by machine learning to identify the low-quality read segments to be scrubbed. We overcame several challenges inherent in this process. First, read-to-read alignment is a quadratic problem that traditional alignment tools such as BWA and Bowtie are not built to handle efficiently [21]. Second, because the dominant type of error in some long read sequencers is (potentially large) indels [2], exact alignments can be difficult to achieve. A recent method called MiniMap2 [22] addresses both of these problems by performing read-to-read overlapping by identifying read pairs that share a number of co-linear k-mers called “minimizers” [22, 23]. This avoids the difficult problem of exact alignment and runs over 50 times faster than BWA, making read-to-read comparisons tractable [22]. Finally, because these read overlaps only provide information on a subset of k-mers shared between reads, we are faced with a challenging pattern recognition problem. Namely, how many k-mers in a region of a given query read need to be supported by other reads, and by *how many* other reads, for that region of the query read to be considered high-quality?

We addressed this challenge by using deep learning, a powerful and popular machine learning paradigm [24]. Deep learning has been increasingly applied in recent years to problems within the biological sciences. A recent notable example is DeepVariant, which achieved superior results in variant calling competitions and benchmarks using a deep learning method called Convolutional Neural Networks [25]. In MiniScrub, we developed a novel method for encoding read-to-read overlaps into “pileup” images, with information such as minimizers matched, quality scores, and distance between minimizers encoded in the color pixels of the images. These images were used as input into a Convolutional Neural Network (CNN), which is optimized to detect local patterns such as those present in images [24], to predict which read segments are of low-quality. See the Methods section below for an explanation of these terms. We show in the Results section that scrubbing with MiniScrub is able to robustly improve read quality and downstream assembly quality, especially in the metagenome setting, even though assemblers already implement a read error correction step.

## Implementation

### Method overview

**Fig. 1:**
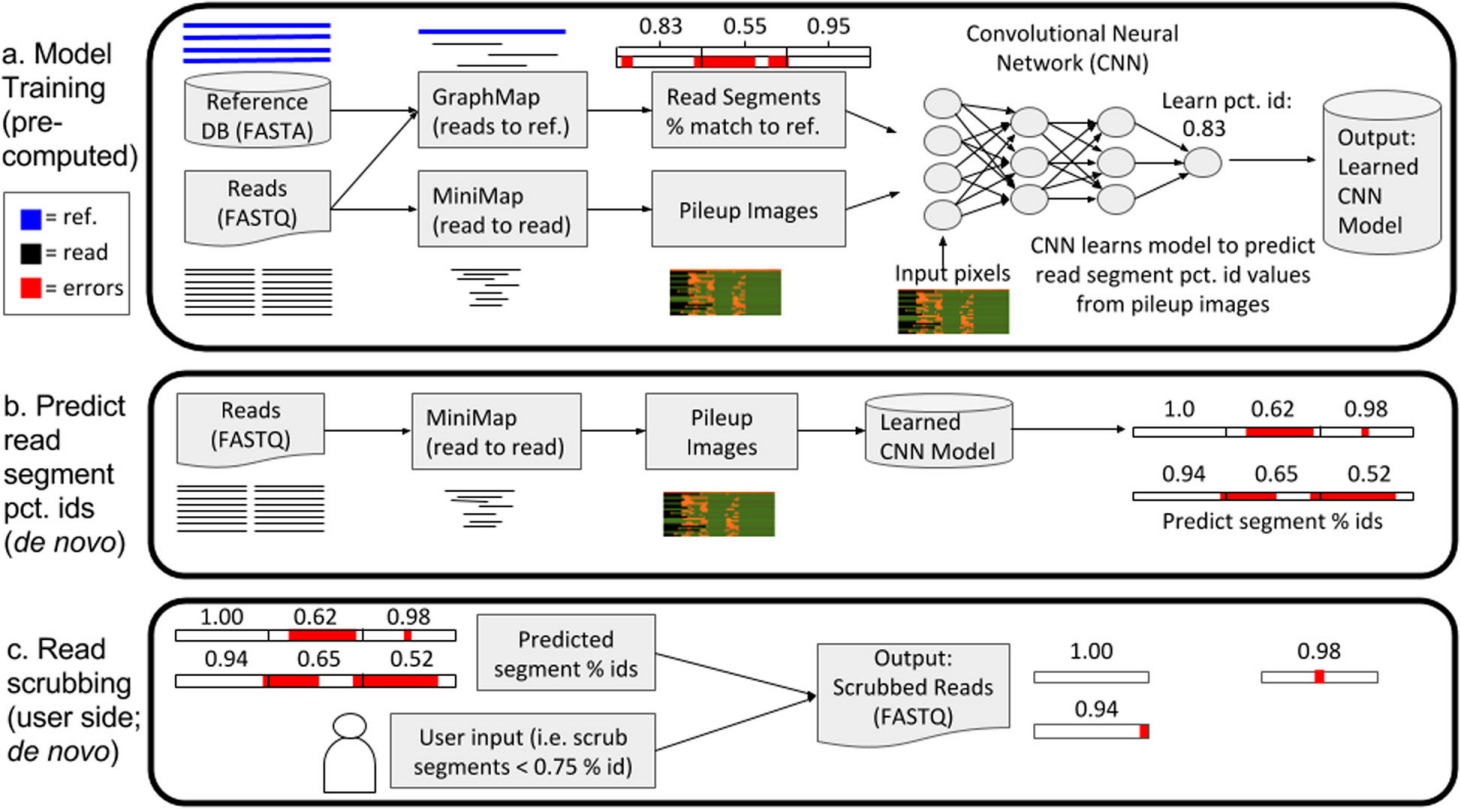
Overview of MiniScrub. The Convolutional Neural Network (CNN) must be trained to predict sequence segment percent identity (percent match to reference) from the read-to-read overlaps. To generate ground-truth percent identity for read segments, reads are generated from known genomes in a reference database, then GraphMap [26] is used to map those reads to the reference, from which we calculate the percentage of bases from each read segment that match the reference genome. We also use MiniMap2 to generate read-to-read mapping, then encode the information into an RGB “pileup” image for each read, which is then split up into shorter segments. We then train the CNN to learn the segment percent identity from the pileup images and save the model. On the user side, users run MiniMap2 on their set of reads and specify a cutoff threshold for read segments to scrub. The learned CNN model then predicts read segment percent identity and scrubs the segments below the quality threshold, outputting a new FASTQ file with the scrubbed reads

The three steps involved in MiniScrub are illustrated in Fig. 1 and explained in further detail in the subsections below. The first step is training a CNN model, a step only needs to be done once, in order to learn the error profile of a certain sequencing technology and base caller. The learned model can then be applied to any dataset of the same sequencing technology and basecaller that it was trained on. The model training step starts with building a training set with reads from a known reference genome. These reads are mapped using GraphMap [26] to the reference genomes. We then divide a mapped read into short segments, defined by a number of minimizers (see following section). For each read segment we calculate its percent identity, e.g. the percentage of bases in the read that match the reference, as labels. Note that matching to the reference is only used here because this is the training stage; reference genomes are not needed in the de novo application stage. We then use a modified version of MiniMap2 [22] to obtain read-to-read overlaps between all reads in the training set (see below for details), and embed relevant information (minimizers matched, distance between minimizers, and base quality scores) into Red-Green-Blue (RGB) pixels to form “pileup” images. One image is generated for each read, and is then broken into the same short segments as above.

A CNN model is then trained with the above data, learning a mapping from a pileup image of a read segment to the percent identity of that read segment. This process is explained more in the subsections below. After the training phase, users can use MiniScrub to generate images and segments of reads from the same sequencing technology, and predict the percent identity of each read segment. Reference genomes are only needed for generating the labels (percent identify of a segment) in the training phase so that the CNN can learn the relationship between how much a read is supported by other reads (represented in pileup image form) and the accuracy of that read. When subsequently presented with pileup images from a dataset that may have novel sequences or for which a reference database is unavailable, MiniScrub’s pre-trained CNN will be able to de novo predict the accuracy of reads based on the relationship between pileup image and accuracy that it learned in the training stage.

Finally, users can scrub out the segments below a user-set percent identity threshold (e.g. 0.8). Taking a FASTQ file as input, reads are split after low quality segments are removed, and they are written into a new FASTQ file.

### Read overlapping using MiniMap2 and minimizers

We use MiniMap2 to rapidly obtain all-to-all read overlaps [22] as it is efficient and robust to indels. MiniMap2 is based on identifying reads that share many co-linear “minimizers” [23]. Briefly, minimizers are the k-mers out of a set of *w* consecutive *k*-mers that minimize a certain function (such as alphabetical order). If two reads share the same *w* consecutive *k*-mers, they are guaranteed to share the same minimizer at that position; thus the minimizers shared between reads are an effective compressed representation of how closely reads match each other. We modified the MiniMap2 program to output the positions of all minimizers of all pairs of reads. Intuitively, if a minimizer in a given read is supported by many other reads, then there is a high likelihood that those k bases covered by the minimizer are error-free, while if no other reads covering the same sequence share that minimizer, it is likely to contain an error. For more details on minimizers, see the original paper by Roberts et. al [23].

### Pileup image generation and deep learning with CNNs

Since CNNs are best adapted for image input, we developed a method for generating images from the read overlaps, which we refer to as “pileup” images [16]. One “pileup” image was generated for each sequencing read, since MiniMap2 uses each read as a “reference read” once and gathers a set of “matching reads” for each reference read (forming a read “pile”). We randomly choose 24 of the matching reads (including the reference read itself) to generate the pileup image; we observed little gain in performance with more reads.

An example pileup image is shown in Fig. 1. Pileup images are generated by embedding the overlaps between a reference read and its matching reads into Red-Green-Blue (RGB) pixels, forming an image. In the image, each column of pixels represents a minimizer in the reference read. The top row in each image represents the reference read, while subsequent pixel rows represent matching reads, thus each image has 24 rows. For each pixel, the red channel indicates whether or not a read contains this minimizer (yes: value 255, no: value 70). The green channel is the average base quality score doubled such that it ranges from 66-254. The blue channel represents the distance to the next minimizer; intuitively, if the blue pixel value is highly different between the reference read and a matching read, one of them likely has an indel. Finally, a (0,0,0) (black) pixel was entered for a section of a matching read that MiniMap2 did not identify as being part of the match. After the pileup image is generated for a read, it is divided into 48-minimizer-wide segments (segments of the reference read spanning 48 minimizers), meaning each image is 48 pixels long. This value was chosen for a strong balance between resolution and accuracy of predictions, but can be modified by the user.

For training CNN models, we use a modified version of VGG16, named after the Visual Geometry Group at Oxford and the number of layers in the network [27]. We chose VGG16 because it is among the most successful CNN architectures available [28], its architecture is open source [27] and widely implemented, and we view it as general-purpose and not overly-adapted to its original image classification task. The original architecture consists of 13 convolutional layers and three fully-connected layers. Each convolutional layer uses 3 ×3 pixel filters. VGG16 was originally developed to classify an image as belonging to one of 1000 categories, but since we are seeking to predict a real number from 0 to 1 (percent identity), we modified the VGG16 architecture to output a single real value. Even though we adapted the VGG16 architecture, we trained our own model weights from scratch, as we found the open-source VGG weights to be too adapted to their original image classification task to work well for our purposes.

We experimented with several optimizers, learning rates, and other hyperparameters. Empirically, we found that the Adam optimizer [29] with a learning rate of 0.0001 and mean squared error loss worked well. Weights were initialized using the Glorot uniform initialization [30] and the network was trained for five epochs. The code in the linked BitBucket repository has further details.

### Datasets, hardware, and software

We evaluated the performance of MiniScrub on two Oxford Nanopore datasets, which we refer to as the “Low Complexity” or “LC” dataset and the “High Complexity” or “HC” dataset. The LC dataset is used in most of our analyses, while the HC dataset is used in this section to evaluate cross-dataset performance. The LC dataset consists of two species sampled at high coverage, *Escherichia coli* (204 × coverage) and *Sphingomonas koreensis* (140 × coverage). In total, the LC dataset contained 747,598 reads averaging 2.6kb in length, out of which 724,140 were successfully mapped to the reference genomes. The HC dataset consists of 260,930 reads sampled from 26 different species, at a much lower coverage (0.005 × to 64 ×). The composition of the HC dataset is explained further in [31] and both datasets are available via the National Energy Research Scientific Computing Center (NERSC) cloud (see the BitBucket repository linked in the abstract). Both datasets were sequenced with Oxford Nanopore MinION flowcell FLO-MIN107 and were basecalled with Albacore version 1.2.1. We recommend users to train new models for new flowcell and base caller versions.

The hardware used in the study was an NVIDIA DGX-1 deep learning system, which has 8 Tesla V100 GPUs, 128GB GPU Memory, 512GB System memory, 40,960 CUDA cores, 5,120 NVIDIA Tensor Cores, and a Dual 20-Core Intel Xeon E5-2698 v4 2.2 GHz Processor. However, only a small fraction of these resources were ultimately needed by our experiments, and GPUs are not required to run MiniScrub, though MiniScrub will be much slower without them. All experiments were performed with MECAT version 1.3, Canu version 1.7, TensorFlow version 1.8, and Keras version 2.2, with the exception of one Canu run with version 1.6, noted in the results section. Because MiniScrub has many dependencies, we also created two docker images, one GPU-based and one CPU-based, for users who do not wish to build from source.

## Results

### MiniScrub robustly predicts low-quality segments within Nanopore reads

MiniScrub predicts the “percent identity” (percent of correct bases) of each segment of a read (defined by a number of bases or minimizers) and scrubs out segments below a user-set threshold, splitting the reads at the low-quality regions. To evaluate its performance, we use the Mean Squared Error and Pearson and Spearman correlations between the predicted percent identity by MiniScrub and the actual percent identity recovered from mapping the reads to the reference. Given our suggested user cutoff of 80% identity (or 0.8), we also calculated the sensitivity and specificity of MiniScrub’s ability to retain high-quality segments. In this case, high sensitivity translates into a low false negative rate, which is desirable as we should retain the high-quality segments as much as possible.

First, we evaluated MiniScrub’s performance by training its model on 25,000 reads, for 5 epochs, from the LC dataset (Methods) and tested its performance on the remaining reads. The results indicated that MiniScrub accurately predicted percent identity of read segments, with a Mean Squared Error of 0.003 and Pearson/Spearman correlation of 0.827/0.805 between the predicted and actual percent identities. Furthermore, given a user-specified cutoff of 0.8, MiniScrub had 95% sensitivity and 68.1% specificity, meaning that it retained 95% of read segments that were actually above the 0.8 threshold and successfully removed 68.1% of those below. This is a conservative setting, and more cutoff parameters can be tuned to scrub more aggressively.

We next assessed the performance of MiniScrub using two datasets generated from the same sequencing technology and base caller using the above metrics, to ensure that MiniScrub does not overfit to a single dataset. In contrast to the highly-covered, low-complexity community of *E. Coli* and *S. Koreensis* in the LC dataset, the HC mock community consists of 26 species at much lower average coverage, representing a very different application setting (Methods). We tested four different settings: training MiniScrub on the LC data and testing on the LC data, training on LC and testing on HC, training on HC and testing on LC, and training on HC and testing on HC. We ran MiniScrub for each setting by training the CNN on 25,000 reads from the training dataset for 5 epochs, and calculated the mean squared error, Pearson correlation, Spearman rank correlation, and sensitivity/specificity at a 0.8 cutoff threshold on 5,000 images randomly drawn from the testing dataset. These results are shown in Table 1; note that the first column corresponds to the experiment described in the previous paragraph.

**Table 1:** Results from training and testing on different datasets

	LC train, LC test	LC train, HC test	HC train, LC test	HC train, HC test
Mean Sq. Error	0.00300	0.00447	0.00312	0.00391
Pearson	0.827	0.747	0.809	0.772
Spearman	0.805	0.795	0.778	0.802
Sensitivity	0.950	0.891	0.938	0.889
Specificity	0.681	0.734	0.681	0.751

“LC” is a low complexity, high coverage (140 × to 204 ×) community derived from 747,598 reads from only two species, *Escherichia coli* (204 × coverage) and *Sphingomonas koreensis* (140 × coverage). “HC” is a high complexity, low coverage (0.005 × to 64 ×) community derived from 260,930 reads from 26 species, described in [31]. The cutoff point for the sensitivity/specificity results was set at 0.8. We use the notation “LC train, HC test” to mean training the model on the LC data and testing it on the HC data

We observed comparable Spearman correlation across all settings, while models tested on the HC data trade off some sensitivity for higher specificity and have slightly worse Mean Squared Error and Pearson correlation. The small difference is likely due to the presence some low-coverage genomes in the HC data, as low-coverage reads will be less discriminatively scrubbed because they have less support from other reads. The prediction accuracy is comparable regardless which dataset is used for training, suggesting that MiniScrub recognizes the error patterns shared by these two different datasets.

### Scrubbing enriches the high-quality read population

**Fig. 2:**
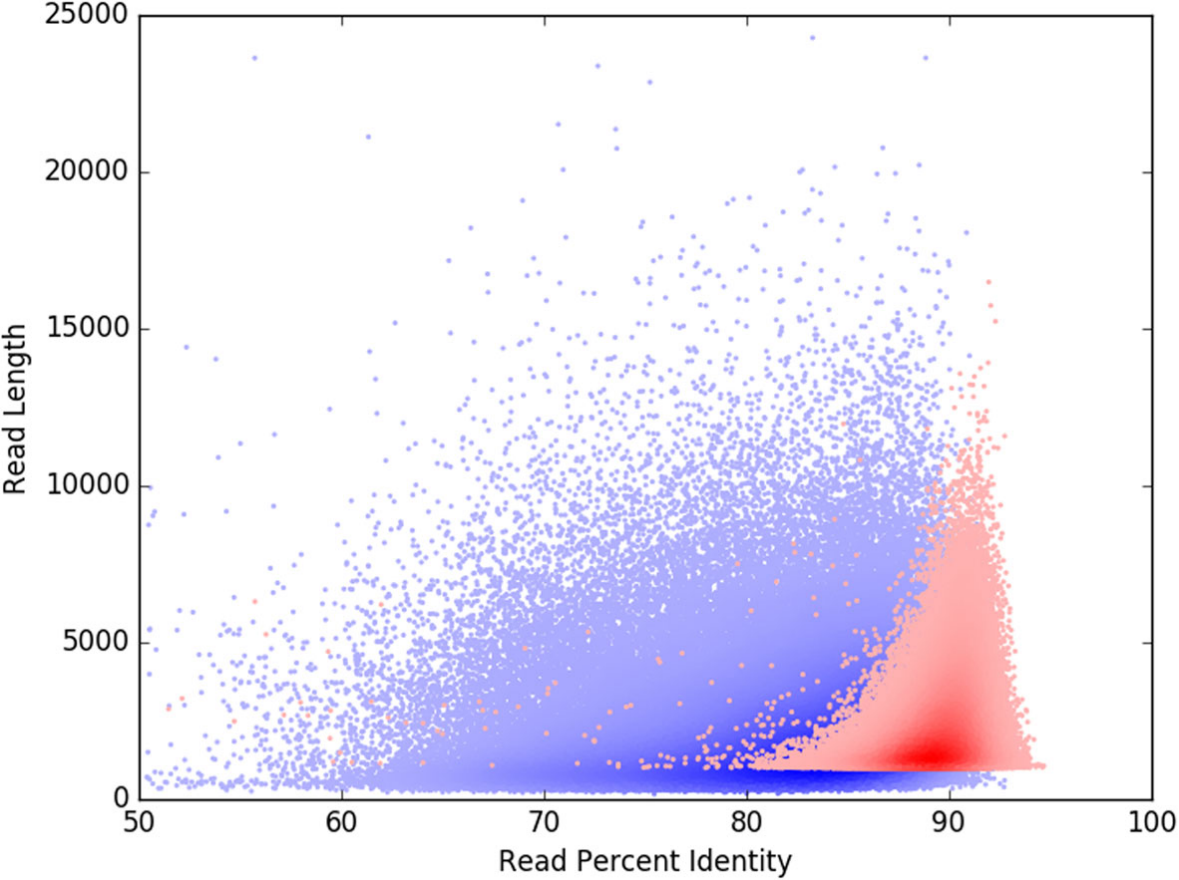
Density scatter plot showing average read quality improvement by MiniScrub versus raw reads. The X-axis shows read percent identity to the reference while the Y-axis shows read length. Raw reads are in blue while scrubbed reads are in red. The darkness of the color indicates increased “density” – more reads fall into a darker region of the graph than the lighter areas. MiniScrub scrubs out most of the low-quality segments in low quality reads while leaving high quality reads intact, increasing average read percent identity by over 3%, from 83.1 to 86.2%. Average read length decreased from 2673 bases to 1594 bases due to splitting reads where low-quality segments were removed. Reads > 25kbp have low density, and are not shown in order to keep the substantive portion of the graph relatively large

To test whether or not scrubbing improves read quality, we compared the reads from the LC dataset (Methods) before and after scrubbing by aligning them to the reference genome to obtain percent identity. As shown in Fig. 2, after scrubbing we observed significant improvements in the read quality. First of all, the majority of the reads with a percent identity between 60-80 have been scrubbed, resulting in more, shorter reads between 85-95 percent identity. Even though MiniScrub does not perform error-correction, scrubbing out a small percentage of low-quality regions (presumably chimera junctions or large indels) nevertheless raises average read percent identity by over 3% (from 83.1 to 86.2%). As shown in Table 1, MiniScrub retains 95% of high-quality read segments (sensitivity); this is reflected in Fig. 2, as most of the reads with high percent identity remain similar in length. Overall, average read length after scrubbing was reduced from 2673 to 1594 bases, while the median was reduced from 1973 to 1161 bases.

### MiniScrub improves read error correction in the metagenome setting

MiniScrub is intended to be used as a preprocessing tool that can improve downstream analysis. Due to the high error rate of long reads, error correction is often performed before other tasks such as assembly or structural variation detection. We tested whether MiniScrub could be applied before read error correction to improve its performance. In particular, popular read error correction methods, such as the error correction step in Canu [19], are developed with the single-genome setting in mind and may not work well when multiple genomes are present in the sample. To investigate this setting, we applied Canu’s error correction step to the high complexity (HC) dataset [31] both with and without scrubbing the reads beforehand, and then aligned the corrected reads to the source genomes with GraphMap [26]. Results are shown in Table 2.

**Table 2:** MiniScrub improves read error correction in the metagenome setting

	No scrubbing	With scrubbing
Avg. coverage pct.	47.04%	**52.71%**
Avg. mean coverage depth	3.08	**3.34**
Pct. of genomes above 1.0 coverage depth	46.67%	**60.00%**

Reads from the high complexity (HC) dataset [31], both with and without scrubbing beforehand, were corrected using Canu’s [19] error correction module and then aligned to their reference genomes with GraphMap [26]. The statistics in the table are averages across all source genomes that had non-zero coverage. Applying scrubbing before read error correction improves average coverage percentage and the average mean coverage depth across the source genomes, and leads to a larger number of source genomes having a mean coverage depth of at least 1.0 Best performance numbers are shown in bold

Applying read scrubbing before read error correction led to improvements in average coverage percentage and coverage depth for the genomes in the HC dataset. The average coverage percent of the source genomes increased from 47.04 to 52.71%, the average mean coverage depth across source genomes increased from 3.08 to 3.34, and the percentage of genomes with a mean coverage depth of at least 1.0 increased from 46.67 to 60%.

### MiniScrub leads to improvements in speed and/or accuracy of de novo assembly

We tested whether or not MiniScrub can be used as a preprocessing step to improve de novo assembly. Several recent long read assembly methods for Nanopore have been developed, including Canu [19], MECAT [32], DALIGNER [21], and more. We chose Canu (version 1.6) and MECAT (version 1.3) for this experiment, as Canu is a popular and well-established method, while MECAT is a newer method that is similar to Canu except with one of the slowest steps of Canu optimized to be faster [32].

We assembled the LC dataset with MECAT and Canu using either raw reads or scrubbed reads. MECAT seems to have problems with very long reads, so we split raw reads longer than 100kb into 100kb segments for it to run without errors. Twenty six reads were split into 67 segments in this manner. MiniScrub by default removes reads shorter than 500 bases, but Canu had problems with reads below 1kb, so for the Canu test, we instead excluded reads shorter than 1kb. Results were evaluated using Quast [33] and are shown in Table 3.

**Table 3:** MiniScrub reduces downstream assembly errors

	MECAT Raw	MiniScrub + MECAT	Canu Raw	MiniScrub + Canu
% genome assembled	79.39%	**99.86%**	99.69%	**99.71%**
NGA50	242478	**1053459**	**1055037**	696460
LGA50	12	**3**	**2**	5
# of contigs	38	**11**	**7**	19
# mis-assembled contigs	28	**5**	2	2
# local mis-assemblies	209	**4**	5	**3**
# indels > 5 bp	1099	**394**	84	**46**
Runtime (hours)	**2.5**	9	80	**9**

MiniScrub significantly improves assembly, tested with MECAT [32], increasing genome coverage and NGA50 while limiting LGA50, mis-assemblies, mismatches, and indels. Canu’s assembly had slightly reduced errors and misassemblies when reads were preprocessed with MiniScrub, but the assembly was more fractured, likely due in part to resolving large misassemblies and indels. Notably, Canu assembly of raw reads took about 3.5 days, while the MiniScrub+Canu pipeline took about 9 hours, likely due to a reduction in the amount of error correction needed in the latter situation. Results were evaluated using QUAST [33] Best performance numbers are shown in bold

After scrubbing the reads, MECAT assembly quality was dramatically improved, with genome coverage increasing from about 79.39 to 99.86%, mis-assembled contigs decreasing from 28 to 5, local mis-assemblies decreasing from 209 to 4, and the number of indels longer than 5bp reduced from 1099 to 394. NGA50 and LGA50 measure the size and number of correctly-assembled contigs required to cover half of the reference genome, with contigs taken in descending order by length. Concretely, MECAT assembly with raw reads required 12 contigs with size 242,478bp or longer to cover half of the reference genome, while assembly with the scrubbed reads only required 3 contigs, which were all 1,053,459bp or longer. Thus, the scrubbed reads produce an improved assembly with fewer, longer contigs that have fewer mis-assemblies and cover much more of the reference genome. Notably, MECAT applies an error correction step [32], so MiniScrub significantly improves performance as a preprocessing step even when subsequent read error correction is performed. This illustrates the potential of using read scrubbing, read error correction, and assembly in tandem.

The difference between Canu assemblies with raw reads and scrubbed reads is much smaller compared with MECAT assemblies. Scrubbing reduces local misassemblies from 5 to 3, and from 84 large indels to 46, while the assembly becomes slightly more fragmented. Scrubbing still improves the percentage of the genomes assembled, indicating that the removed sequences that caused fragmentation were low-quality or redundant. Notably, Canu runtime was dramatically reduced on scrubbed reads, decreasing from over 3.5 days on raw reads to 9 hours on scrubbed reads, including the read scrubbing step. This is probably due to a large amount of low-quality data being removed, simplifying the error correction step. In contrast, MECAT was much faster, taking about 2.5 hours with raw reads, but about 9 hours to scrub the reads and run assembly. This suggests that the dataset could be assembled quickly and accurately using either MiniScrub+MECAT or MiniScrub+Canu, but without scrubbing the assembly could be inaccurate or time-consuming.

### MiniScrub’s performance across different parameter settings

By default, MiniScrub has a default pileup image size of (Length, Depth) = (48, 24), meaning 48 minimizer-wide segments, and up to 23 matching reads for each query read. Additionally, MiniScrub uses minimizers with settings (w,k) = (5,15), meaning that a minimizer k-mer of length 15 is selected out of each 5 consecutive 15-mers. We sought to evaluate whether MiniScrub was effective under these default parameter settings, and whether it would be robust to reasonable adjustments to these parameters. Starting from the default settings of (Length, Depth) =(48, 24) and (w,k) =(5,15), we varied each pair of parameters in turn while holding the other pair constant. Namely, we evaluated the settings of (Length, Depth) = (36, 36) and (w,k) =(7,17). We ran MiniScrub for each setting by training the CNN on 25,000 images from the LC dataset for three epochs and calculating the mean squared error, Pearson correlation, Spearman rank correlation, and sensitivity/specificity at a 0.8 cutoff threshold. These results are shown in Table 4, along with results from the default parameters for comparison. As the table shows, MiniScrub performs robustly under all tested parameter settings, giving similar performance. The results also demonstrate how a user can adjust sensitivity and specificity performance to their needs by modifying parameter settings. For example, see the increased performance in specificity for the “(w,k) =(7,17)” column, at the cost of some sensitivity.

**Table 4:** Performance with different parameter settings

	Default	(Length, Depth) = (36, 36)	(w,k) = (7,17)
Mean Sq. Error	0.00300	0.00329	0.00305
Pearson	0.827	0.821	0.830
Spearman	0.805	0.786	0.810
Sensitivity	0.950	0.934	0.914
Specificity	0.681	0.693	0.780

Performance with different parameter settings. w and k refer to the minimizer parameters, while Length and Depth refer to the length and depth of each pileup image segment, which correspond to the number of minimizers in that read segment and the number of matching reads used. The default settings are (w,k) =(5,15) and (Length, Depth) = (48, 24). The columns show the performance when varying one of these settings and with cutoff 0.8

## Discussion

We developed a method called MiniScrub that performs de novo long read scrubbing using the combined power of fast approximate read-to-read overlapping, deep Convolutional Neural Networks, and a novel method for pileup image generation. We demonstrated that it accurately scrubs out low-quality segments within Nanopore raw reads to improve overall read quality, and that the scrubbing improves read error correction in the metagenome setting. We also highlighted one particular application area, de novo assembly, where results can be improved by applying MiniScrub as a preprocessing method.

We show that scrubbing facilitates downstream read-correction process, improving both overall read quality and genome coverage in the metagenome setting. This may be primarily due to improved coverage of several low-coverage genomes. The genomes in the HC dataset vary significantly in coverage. In this dataset, Canu may over-correct error-prone reads from low-coverage genomes in favor of high-coverage genomes, since Canu expects only one source genome. By scrubbing the reads beforehand, the remaining read segments for the low-coverage genomes are higher-quality and more consistent with each other, and are thus less likely to be over-corrected by Canu.

Besides de novo genome assembly, we expect read scrubbing may also improve other downstream analyses, such as large structural variation detection. As MiniScrub uses a generic framework, it is possible that MiniScrub can learn technology-specific error profiles. Even though we focused on Oxford Nanopore reads in this study, read scrubbing may work on other long read technologies, such as PacBio SMRT. One would have to train a new CNN model for each different sequencing technology.

As MiniScrub splits reads at the point of scrubbing (chimera junctions or indels), splitting at indels will lead to lower assembly contiguity, especially affecting the low-coverage regions. Even though this may be a trade-off between contiguity and fewer errors, this leaves room for future improvements. One of the potential improvements would be to train the model to discriminate the chimera junctions and indels, and only split the chimeric reads while leaving those with large indels for read correction modules to fix.

In our current CNN model, both convolution and pooling are locally performed for small patches of the pileup images separately, without considering contextual dependencies between different patches. An interesting methodological direction would be to change our model to a Convolutional Recurrent Neural Network (CRNN) by adding Recurrent Neural Network (RNN) layers to learn contextual dependencies among sequential data through the recurrent (feedback) connections. This CRNN model may further enhance the predictive performance, especially the ability to detect low-quality regions.

## Conclusions

MiniScrub is a novel deep learning method for improving Nanopore read quality. MiniScrub uses minimizers to quickly overlap long reads, encodes these overlaps into pileup images, and uses a convolutional neural network to predict parts of reads below a certain quality threshold that should be removed. We show that applying MiniScrub robustly improves read quality and error correction and that this improvement leads to a reduction in long indels and local mis-assemblies in downstream assembly. MiniScrub was tested on Nanopore data, but should in principle be generalizable to any long read data, if trained properly. We propose MiniScrub as a novel de novo long read preprocessing tool with particular usefulness in the metagenome setting that can benefit downstream analysis such as assembly. MiniScrub is open-source and available on BitBucket at https://bitbucket.org/berkeleylab/jgi-miniscrub.

## Availability and requirements

**Project name**: MiniScrub

**Project home page**: https://bitbucket.org/berkeleylab/jgi-miniscrub

**Operating system(s)**: Platform independent

**Programming language**: Python 3

**Other requirements**: TensorFlow, Keras, numpy, scipy, matplotlib, pandas, pillow, h5py, scitkit-learn, MiniMap2. Alternately, use one of the docker images as documented on the BitBucket page.

**License**: BSD 3-clause

**Any restrictions to use by non-academics**: None

## Data Availability

MiniScrub is open-source software and the source code is available via BitBucket at https://bitbucket.org/berkeleylab/jgi-miniscrub. In addition to the source code, docker images are also available, as documented on the BitBucket page. Datasets are available via the National Energy Research Scientific Computing Center (NERSC) cloud, with links in the BitBucket.

## Abbreviations

CNN: Convolutional neural network
